# Considerations for the Use of Alloplastic Temporomandibular Joint Replacement in Irradiated Patients: Report of an Off-Label Indication

**DOI:** 10.3390/jcm12206612

**Published:** 2023-10-19

**Authors:** David Faustino Ângelo, Francesco Maffia, Marcus Teschke, David Sanz, Marta Galrito, Henrique Cardoso, Rute Marques, Carlos Nabuco

**Affiliations:** 1 Instituto Português da Face, 1050-227 Lisbon, Portugal; david.sanz@ipface.pt (D.S.); mgalrito@yahoo.com (M.G.); henrique.cardoso@ipface.pt (H.C.); rute.marques@ipface.pt (R.M.); ce.nabuco@gmail.com (C.N.); 2 Faculdade de Medicina, Universidade de Lisboa, 1649-028 Lisbon, Portugal; 3 Centre for Rapid and Sustainable Product Development, Polytechnic Institute of Leiria, 2430-028 Marinha Grande, Portugal; 4 Maxillofacial Surgery Unit, Department of Neurosciences, Reproductive and Odontostomatological Sciences, University of Naples “Federico II”, 80131 Naples, Italy; 5 Independent Researcher, 28195 Bremen, Germany; marcus.teschke@me.com

**Keywords:** temporomandibular joint, head and neck neoplasms, total joint replacement, adjuvant radiotherapy, free tissue flaps

## Abstract

Background: Custom-made alloplastic temporomandibular joint replacement (ATMJR) is not validated in irradiated patients. However, in specific situations, after previous reconstructive surgical failures, the authors hypothesized the role of a customized ATMJR after radiotherapy. Methods: A 65-year-old male patient was referred to Instituto Português da Face—Lisbon, Portugal—after failed attempts of mandibular reconstruction secondary to oral carcinoma resection and partial hemi-mandibulectomy plus radiotherapy of 60 total Grays. Primary reconstruction was performed with fibula free flap. Due to failure, secondary reconstructions were performed with osteosynthesis plate without success. The patient was unable to have adequate mastication and deglutition due to a severe crossbite. The authors treated the patient with an extended customized alloplastic temporomandibular joint replacement (F0M2). Results: With 3 years of follow-up, the patient showed an improvement in masticatory function, mandibular motion, pain levels, and overall quality of life. No complications were observed related to ATMJR. Conclusions: The presented case described how ATMJR, although not a validated option after radiotherapy, can be considered to restore functionality in complex cases with bone and soft tissues problems.

## 1. Introduction

Head and neck cancer (HNC) is the sixth most common cancer in the world. The prevalence of head and neck malignancies is mainly related to tobacco and alcohol use, which are major risk factors of these neoplasms [1]. When diagnosed, most oral cancers are already in an advanced stage, requiring more aggressive treatment. Multiple modalities are often required in the treatment of oral squamous cell carcinoma (OSCC), including major surgery, chemotherapy, and/or radiotherapy (RT) [2]. Tumor resection may include part of the mandibular bone, causing the loss of mandible continuity, architectural distortion, and functional loss, with the possibility to also involve the temporomandibular joint (TMJ) [3,4]. Various options are available to perform immediate mandibular reconstruction, including bone-harvested free flaps, locoregional flaps, and reconstruction with titanium plates [3]. After reconstruction, most patients are submitted to radiotherapy. Severe consequences can arise in the postoperative phase: one of the most common is osteoradionecrosis (ORN), especially in patients with advanced-stage tumors and those exposed to prolonged treatments [5]. ORN-affected bone tissue can be complicated by infections, requiring continuous medications and treatments, reducing the quality of life [2]. In these cases, resection of necrotic areas can be necessary, resulting in further significant bone and soft tissue loss. Several studies have recommended further free flaps as the first reconstruction option for patients with major mandibular defects secondary to osteoradionecrosis [6]. However, when free flaps fail, a salvage reconstruction can be accomplished, with a high risk of poor and unsatisfactory results [7,8]. Alloplastic temporomandibular joint replacement (ATMJR) can offer a comprehensive solution for individuals who have undergone several surgeries and still suffer from significant functional impairment and pain [9]. This case report aims to describe a challenging case of an irradiated patient submitted to several failed mandibular reconstruction surgeries and successfully treated with an extended customized alloplastic temporomandibular joint replacement (F0M2—Higginson Classification) [10].

## 2. Materials and Methods

### 2.1. Case Presentation

**Figure 1:**
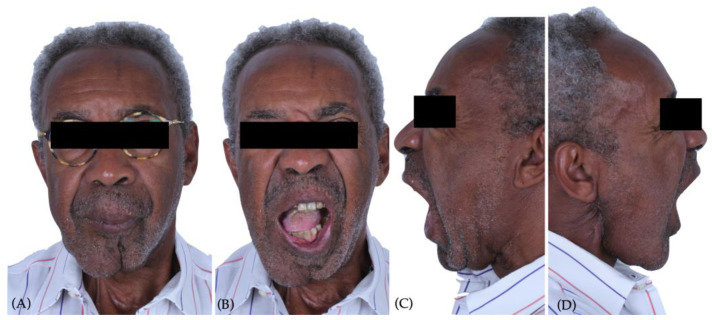
Preoperative clinical examination: (**A**) frontal view of the patient in rest position showing right hemifacial deformity; (**B**) frontal view of the patient during maximum mouth opening presenting right sever mandibular deviation; (**C**) left side maximum mouth opening; (**D**) right side maximum mouth opening, showing severe fibrosis in the right mandibular angle, submandibular and neck region.

In 2020, a 65-year-old male patient was referred to Instituto Português da Face after several attempts of mandibular reconstruction due to oral cavity carcinoma (Figure 1).

**Figure 2:**
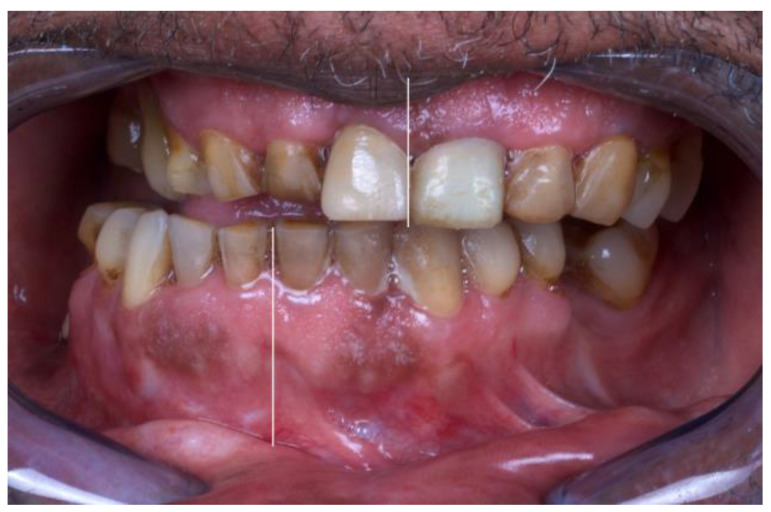
Preoperative occlusion: severe cross-bite with deviation of the interincisal dental midline to the right side.

The patient presented with a severe right crossbite (Figure 2), inability to feed due to absence of proper mastication, with a dietary score of <2/10 (liquid scores 0, full diet scores 10), with a recent weight loss of more than 10 kg, achieving 63 kg of weight, with a maximum mouth opening of 24 mm (measured with a certified mouth ruler), and a chief complaint of pain in the temporomandibular area (visual analog scale, VAS, 7/10).

**Figure 3:**
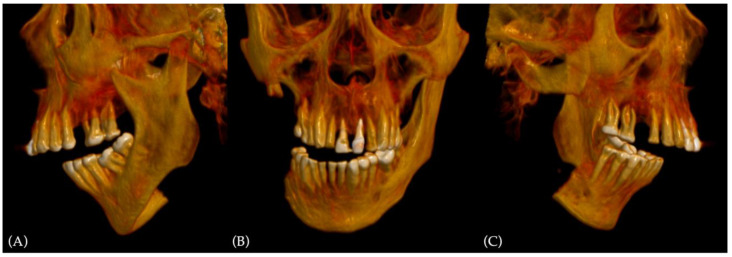
Preoperative 3D CT scan reconstruction showing preoperative malocclusion and the bone defect: (**A**) left-side view; (**B**) coronal view, with severe deviation to the right side; (**C**) right-side view, displaying the amount of bone missing from the different previous surgery.

In his past clinical history, the patient was submitted to several reconstruction surgeries after ablative surgery of head and neck squamous cell carcinoma (HNSCC) of the mandibular trigone. The first surgery was tumor resection with TMJ-sparing partial hemimandibulectomy, associated with elective neck dissection (levels I-III) and primary reconstruction with the left fibula free flap. The pathologic staging was pTNM T4N0M0. After surgery, in March 2018, the patient was submitted to RT for two months, with 60 Grays (Gy) on the surgical site and more than 40 Gy on the right lymph nodes. A cause of osteoradionecrosis developed in the mandibular area around the resection, a surgical debridement of the fibula flap was required one month later. Further clinical evaluation revealed parotitis owing to RT and exposed mandibular bone spikes. Parotidectomy and mandibular osteotomy with total free flap removal were carried out, followed by reconstruction with osteosynthesis plate. A new local infection was established, and the patient was re-operated in 2019 for purulent material debridement an osteosynthesis plate removal, leaving a huge bone defect (Figure 3).

In the preoperative planning stage, indications and contraindications were balanced: on the one hand, occlusal instability, daily difficulty in chewing and poor feeding (dietary score < 5/10), VAS pain score > 5/10 (7/10), and several failed reconstructions indicated ATMJR; on the other hand, history of osteonecrosis with repeated infections, reduced bone quality and poor soft-tissue quality represented contraindications.

In August 2020, a decision was taken for a final reconstruction attempt at Instituto Português da Face, where the main surgeon proposed a reconstruction with an extended custom-made ATMJR.

### 2.2. Custom-Made Prosthesis Manufacturing

**Figure 4:**
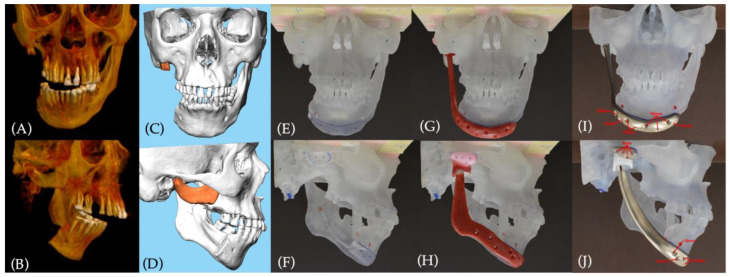
Virtual surgical planning and custom-made temporomandibular joint prosthesis manufacturing: (**A**,**B**) coronal and right-side view of the preoperative CT scan 3D-reconstructed model in current malocclusion; (**C**,**D**) coronal and right-side view of the 3D model in planned transferred occlusion, with planned bone removal: condyle and coronoid process; (**E**,**F**) frontal and right-side view of the 3D-printed model with planning marks before prosthesis simulation model elaboration; (**G**,**H**) frontal and right-side view of the 3D-printed model with prosthesis plastic simulation model; (**I**,**J**) frontal and right-side view of the 3D-printed model with final version of the manufactured prosthesis: attention was made in the development of a smoothed mandibular angle. Red arrows in I-J indicate the details for screw.

The alloplastic temporomandibular joint replacement was planned virtually in collaboration with Stryker (TMJ Concept, Stryker, Portage, MI, USA). The preoperative CT scanning protocol was followed adopting the standardized TMJ Concept helical scanning parameters: the scan area included TMJ, mandible and maxilla; the acquisition algorithm was standard; the CT field of view (FOV) was adjusted to 22 cm, with a pitch of 1:1; slice interval and slice thickness were adjusted to 0.5 mm; the scan was executed without contrast and with a Gantry tilt angle of 0°; obtained data were archived in uncompressed digital imaging and communications in medicine (DICOM) image data (Figure 4A,B). After CT scan acquisition, the occlusion was studied with plaster models. Due to the loss of a great amount of bone between the condyle and the mandibular body, the patient was no longer able to occlude properly, showing a persistent deviation of the interincisal dental midline to the right side. Accordingly, plaster models were realised to achieve a transfer of the occlusion to the 3D-reconstructed model by virtual planning. After transferring virtually the wanted final occlusion, the prosthesis development started on the 3D-reconstructed virtual model.

The first step was the analysis of the available bony surfaces, to plan eventual osteotomies. The right TMJ condyle and coronoid process were planned to be removed to create space for the condyle–fossa elements. The bone of the mandibular body was considered clinically and radiologically healed from ORNJ and previous infections, so further mandibular resections were planned (Figure 4C,D).

The 3D-reconstructed virtual model was 3D-printed to materially plan the prosthesis (Figure 4E,F). The first planning included a TMJ Concept prosthesis model, extended to the contralateral mental nerve foramen. A plastic simulation model of the prosthesis was realized to evaluate fitting (Figure 4G,H). During the simulation check, special attention was given to the mandibular angle: since the skin elasticity of the region was reduced due to fibrosis; the decision was to keep the mandibular extension to the contralateral side, but to realize a prosthesis with a smaller lateral projection of mandibular angle, to avoid excessive tension and potential exposure of the implanted material. Once surgeons and engineers validated the final model of the prosthesis, its manufacturing was commissioned and checked again on the 3D-printed model (Figure 4I–J). The different components of the prosthesis were realized in different materials: the glenoid fossa backing was realized in unalloyed titanium, while the condylar lodge was made of ultra-high molecular weight polyethylene; the mandibular component was realized in cobalt–chromium–molybdenum for the condylar head, while the mandibular ramus and body were realized in titanium alloy. Osteosynthesis was planned to be performed with n°4 2.0 system 6 mm self-tapping screws in the glenoid fossa component, and with n°6 2.0 system 16 mm and n°2 2.0 system 14 mm self-tapping bicortical screws for the mandibular component.

Following the Higginson classification, the custom-made prosthesis was composed of F0-M2/fossa component F0, and standard fossa component (contained within fossa)/mandible (ramus) component M2, extended proximally to the contralateral mental foramen [11].

### 2.3. Surgical Technique

**Figure 5:**
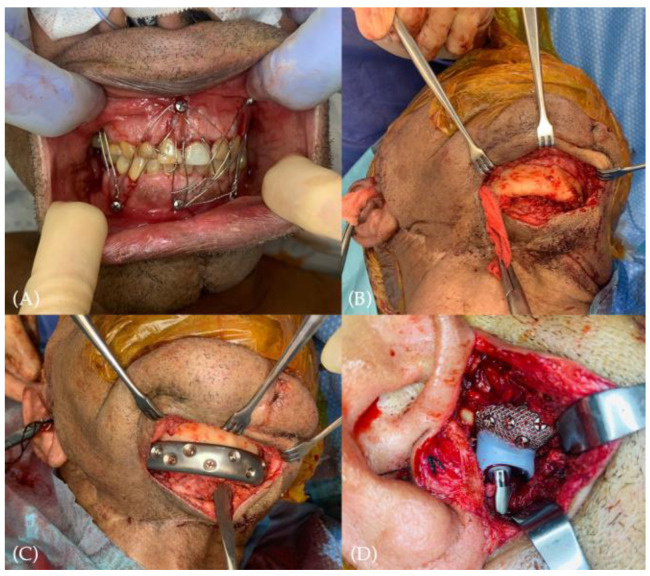
Main surgical steps: (**A**) patient positioned in occlusion with inter-maxillary fixation; (**B**) dissection conducted from the submental area to the preauricular region by blunt tools (gauze showing the connection); (**C**) passage and fixation of the mandibular component in the partially fixed fossa component; (**D**) after confirmation of the correct condyle/fossa relation, fixation with all screws of all the elements.

Before entering the operating room, the patient performed oral rinses with chlorhexidine three times for 3 min each. A premedication with 2 gr of Amoxicillin/Clavulanic acid (875 mg/125 mg) + Gentamicin 3 mg/kg was dispensed one hour before surgery. In this specific case, before asepsis, the patient was positioned in occlusion with inter-maxillary fixation (IMF) (Figure 5A).

Face and neck asepsis were performed, drapes were disposed, and mouth isolation was obtained by application of Ioban (3M^®^) antimicrobial drape to reduce the risk of cross-contamination. By preauricular modified endaural incision, condylar and coronoid processes were removed. The glenoid fossa component of the custom-made ATMJR prosthesis (TMJ Concept^®^) was positioned and preliminary fixation to the temporal bone with 3 screws out of 5 for a temporary check. A secondary incision was performed in the submental area to expose the mandibular symphyseal and parasymphyseal areas. The dissection was gently conducted from the submental area to the preauricular region by blunt tools and with systematic irrigation with Gentamicin + Vancomycin (Figure 5B). A special note from the surgeon (D.F.Â.) in this dissection follows: “It was for me extremely difficult to dissect in a fibrotic plan, without mandibular periosteal tube intact, with reduced anatomic landmarks, balancing the dissection without being too much superficial (high risk of prosthesis exposition) and too deep (high risk of oral entrance and cross contamination)”. Once obtained, the connection between the two incisions, the passage of the mandibular component was accomplished, avoiding as much as possible direct manipulation (Supplementary Material: Video S1—ATMJR passage). After confirmation of the correct condyle/fossa relation, the mandibular component was fixed with 4 screws before the final check (Figure 5C,D). IMF was then released, mobility was tested, and occlusion was verified. Thus, all screws were positioned to ensure proper ATMJ prosthesis stability. Incisions were closed by layers.

**Figure 6:**
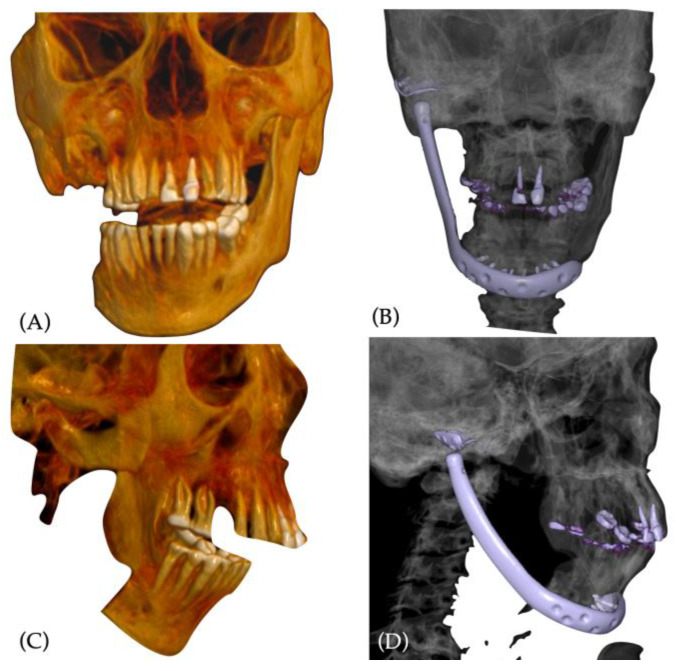
Preoperative and postoperative 3D CT scan comparison: (**A**,**B**) coronal view comparison of the preoperative and postoperative CT scan 3D reconstruction, showing the large bone defect filled by the prosthesis positioning and facial harmonization; (**C**,**D**) right-side view of the preoperative and postoperative CT scan 3D reconstruction showing prosthesis profile and bettering in occlusion.

A postoperative CT scan was performed to check the prosthesis positioning and to have a comparison with the preoperative situation (Figure 6).

### 2.4. Postoperative Evaluation

In the postoperative phase, evolutions in various parameters were assessed during follow-up: mandibular kinetics with maximal mouth opening, mastication, and phonation.

Mastication improvements were evaluated by asking about discomfort related to the texture of food. Three standardized textures were considered: soft (ex: boiled potato, bread loaf) medium (ex: brioche bread, tea biscuits), and hard (uncooked almond) [12].

Speech evaluation included audio and video recording of a brief conversation and of a standard articulation test: vowels (/a/, /e/, /i/, /o/, /u/), consonant phonemes (/p/, /b/, /t/ /d/, /k/, /g/, /f/, /v/, /s/), and percent conversational understandability were assessed.

## 3. Results

### 3.1. Postoperative Phase

**Figure 7:**
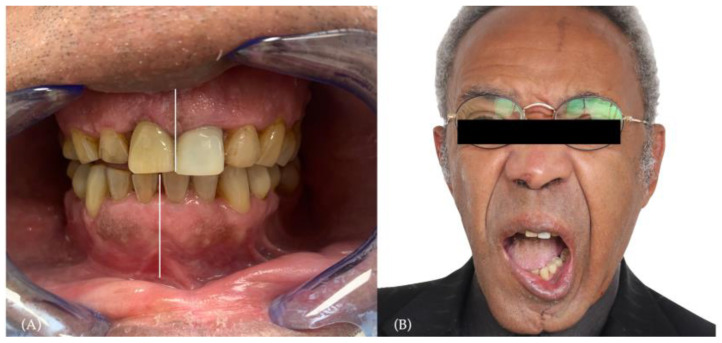
Postoperative phase: (**A**) postoperative occlusion, showing improvement in interincisal dental midline and resolution of the crossbite (**B**).

No complications were observed in the immediate postoperative phase. An improvement in mandibular kinetic was noticeable a few hours after the surgery. In the first weeks after the surgery, the patient presented improvements in facial aesthetics, occlusion, mandibular motion, symmetry, and pain levels (VAS 1/10) (Figure 7).

Gradually, the alimentation was shifted from soft to progressively harder foods, improving the feeding problem. In the months after the surgery, the patient was able to continue to feed himself with a progressive diet, which enabled him to return to his normal weight.

### 3.2. Follow-Up

In the long term, the patient was submitted to routinary check-up every 3 months, resulting from the absence of primary disease. Significant improvements were noticed in functionality. Mandibular kinetic improved, reaching the maximum mouth opening of 30 mm (vs 24 mm preop) and a bettering in symmetrical movements, evaluated by interincisal dental midline shift comparison.

The switch from a liquid diet to a solid diet was considered a success. Considering the texture evaluation, the patient was able to feed himself with food of medium-hard texture. The dietary score increased from 2/10 to 8/10 (liquid scores 0, full diet scores 10).

The speech therapy improved his postoperative phonation and speech intelligibility, increasing gradually the well pronunciation of vowels and consonants.

**Figure 8:**
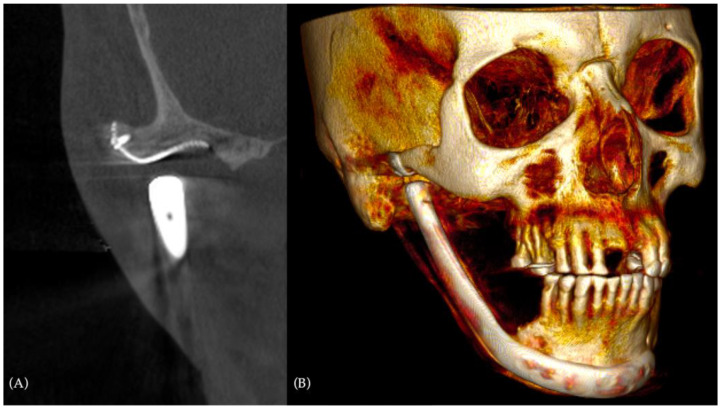
The 3-year-follow-up CT scan: (**A**) detail of the appropriate fitting of the alloplastic fossa to the temporal bone in coronal view; (**B**) three-quarter right view of the CT scan 3D reconstruction, showing stability of the obtained result.

With almost 3 years of follow-up, a complete back to normal life was achieved, with a significant improvement in the overall quality of life. A CT scan at a 3-year follow-up showed perfect fitting of both alloplastic prosthesis elements to the bone (Figure 8).

## 4. Discussion

Ablative surgeries followed by radiotherapy in oncologic head and neck patients can lead to significant complications like radiodermatitis, xerostomia, and osteoradionecrosis [7,13]. Osteoradionecrosis of the jaw (ORNJ) is one of the most debilitating side effects of radiotherapy in patients undergoing treatment for head and neck cancer [2,5]. ORNJ is defined as the process where irradiated bone becomes necrotic and exposed for over three months, with failures in healing, and in the absence of residual or recurrent tumor [7,14]. ORNJ in head and neck cancer patients has variable incidence rates reported in the literature, ranging from as low as 0.4% to as high as 56% [15]. The most prominent risk factor associated with osteoradionecrosis is a high radiation dose: more than 60 Gy is reported as high risk, while 50–60 Gy as intermediate risk [14]. Several classifications tried to organize ORNJ in class and types, the Notani and the Epstein classification are two of the most diffused. The Notani classification is divided in three classes based on clinical examination and orthopantogram, with ORN confined to dentoalveolar bone (class I), ORN limited to dentoalveolar bone or mandible above the inferior dental canal, or both (class II), and class III, including ORN involving the mandible below the inferior dental canal, and adding presence of pathological fracture or skin fistula. The Epstein classification also includes the knowledge of a clinical course describing progressive and non-progressive ORNJ. This classification in organized in the three types resolved: healed (type 1), chronic persistent non-progressive (type 2), and active progressive (type 3). All types can be sub-divided in A and B based on the presence of a pathological fracture [14,16].

In oncological patients submitted to microsurgical reconstruction, severe osteoradionecrosis can lead to free-flap failure, creating the necessity of a secondary reconstruction in an irradiated bone with reduced quality and vascularization [4,13]. A second free flap is reported in the literature as the gold standard reconstruction option for these complex cases [8]. Patients who have undergone radiations appear to have more uncertain surgical outcomes, especially for a second reconstruction free flap [15]. Diffused fibrosis, distorted neck vascularization, necessity of contralateral vascular anastomosis, and reduced bone quality may affect the procedure [8,15]. Thus, in irradiated patients further alternatives to microsurgical reconstruction are needed, and alloplastic replacements can represent a new horizon [9,17].

In our case, the analysis of indications and contraindications to an ATMJR solution was made studying the patient characteristics with the support of total temporomandibular joint replacement guidelines present in the literature. In 2008, A. J. Sidebottom published indications and guidelines for the replacement of temporomandibular joints in the United Kingdom, spreading a first point of reference in the field. Following the indications, total TMJ replacement should be performed after failed conservative management, with a proper diagnosis made with CT scan or magnetic resonance. The pathologies included are disease involving condylar bone loss or damage as degenerative joint disease (osteoarthrosis), inflammatory joint disease, ankylosis, post-traumatic or postoperative condylar loss (including neoplastic ablation), serious congenital deformity, and multiple previous procedures. Specific indications include dietary score < 5/10 (liquid scores 0, full diet scores 10), restricted mouth opening (<35 mm), occlusal collapse, pain score > 5 out of 10 on visual analogue scale, and other issues in quality of life. Contraindications are a local infective process, severe immunocompromise, and severe coexistent diseases [18].

In 2020, the Joint Committee of the Japanese Society of Oral and Maxillofacial Surgeons and the Japanese Society for the Temporomandibular Joint for Clinical Guidelines, proposed an update of the previous indications, adding specific relative contraindications. Analyzing the indication classification, our case matched with postoperative damage, history of multiple invalid surgery, low dietary score, restricted mouth opening, occlusal collapse, and high levels of pain. On the other hand, previous osteoradionecrosis and infections could have represented relative contraindications as reasons of possible poor bone quality, as well as previous local inflammatory conditions and radiation-related fibrosis [19].

In our case, the possible relative contraindications were studied: after experiencing several inflammatory and infective issues, the patient was considered healed from ORNJ; no local inflammatory state was detected in the area despite of dermal fibrosis. Bone quality was considered clinically and radiologically good and stable enough to bear a prosthesis implant without further resections. For these reasons, the patient was evaluated as a proper candidate to receive ATMRJ.

Progressively, bone reconstructions with alloplastic material prostheses are becoming more popular in the maxillofacial surgery field, opening new scenarios and new reconstructive alternatives [6,20]. In temporomandibular joint pathology, ATMJR has been used for the management of end-stage temporomandibular diseases for over 2 decades with success, enhancing mandibular functionality (mastication, deglutition, and phonation) and improving quality of life [9,21,22]. Because of the local inflammatory state post osteoradionecrosis, the quality of the remaining bone, and the high risk of implant exposure due to lack of skin elasticity, ATMJR may not be recommended in irradiated patients because of the higher possibility of failure and severe complications [19,23]. The introduction of virtual surgical planning for temporomandibular joint replacement has extended reconstructive opportunities, fulfilling any reconstructive need and giving the possibility to offer ATMJR in more complex patients as well [9]. Custom-made devices allow a better functional and esthetical satisfying reconstruction for the patient, improving quality of life and clinical outcomes [9,11,21].

An innovative reconstructive option in complex ablative cases is represented by patient-specific spacers: the bone defect after the first ablative surgery is filled with a personalized spacer implant to optimize soft tissue support. It allows keeping distance between bone stumps during radiotherapy and helps having hard tissue continuity for the second stage of the reconstruction (free flap or TMJ prosthesis). Keeping the mandibular periosteal tube intact makes further dissection easier [22].

To the best of our knowledge, this case report is the first to describe an ATMJR implant in a previously irradiated patient who also experienced ORNJ. Since, in the literature, a waiting time of 14 months post-radiation therapy is considered sufficient for the use of alloplastic implants, and the patient’s condition after ORNJ and infections were stable, the authors’ choice was to proceed with implantation of the prosthesis [2].

When compared to other reconstructive techniques such as free flaps, using a TMJ prosthesis shortens surgical time, minimizes secondary morbidity by eliminating the necessity for a donor site, reduces surgical time and consequent risk of infections, reduces inpatient time, and gives instant functionality improvement [6]. Furthermore, this reconstruction technique can be personalized, increasing the quality of outcomes. The possible necessity of surgical revision, prosthesis failure due to loosening of a screw or fracture of the prosthesis due to metal fatigue, a narrow fit of stock prostheses, loss of laterality and protrusion movements due to lateral pterygoid muscle disconnection, and high cost are all potential issues with TMJ prostheses [21,24]. Based on Marcus Teschke’s previous work [22], in the authors’ opinion, the ideal reconstruction in oncologic head and neck patients should be as follows:

1. Virtual surgical planning of the tumor resection and mandibular reconstruction.
2. Digital design of anatomic spacer and custom-made cutting guides.
3. First surgical stage: resection of the tumor and custom-made spacer insertion.
4. Postoperative radiotherapy.
5. Second surgical stage: reconstruction with customized plates with/without free flap and with customized ATMJR if TMJ involvement. Consideration to use ATMJR alone.

## 5. Conclusions

Ablative surgeries followed by radiotherapy in head and neck oncological patients may lead to major complications like facial distortion, loss of function, infections, and osteoradionecrosis of the jaw. RT is an effective but harmful treatment: irradiated bone and soft tissues are associated with reduced blood supply, implicating further complications in the performed reconstruction. Each mandibular reconstruction should be performed only after all these elements have been considered. Fibula free flap is described in the literature as the gold standard reconstruction option for these cases. Nevertheless, other reconstruction options must be considered when FFF fails. ATMJR could be considered as a safe and reliable management modality in selected patients with complex reconstructive needs.

The presented case described how ATMJR, although not a validated option after RT, can be considered to restore functionality in patients irradiated, becoming a viable option in patients with soft and hard tissue problems. Improved quality of life, restored mastication and phonation, reduction of level of pain and increased mandibular kinetic are the main improvements noticed in this reconstruction. Alloplastic prosthesis reconstructions in irradiated bone are not sufficiently described in the literature. Technological evolution positively impacts complex cases such as this one, opening new possibilities. Further studies are necessary to evaluate alloplastic prosthesis role in the head and neck reconstruction surgery.

## Data Availability

The data presented in this study are available in this article.

## Supplementary Materials

The following supporting information can be downloaded at https://www.mdpi.com/article/10.3390/jcm12206612/s1. Video S1: Alloplastic temporomandibular joint replacement: passage and positioning of the mandibular component in the prosthetic glenoid fossa.

## References

[1] Shah J.P., Gil Z. (2009). Current concepts in management of oral cancer—Surgery. Oral Oncol..

[2] Di Carlo S., De Angelis F., Ciolfi A., Quarato A., Piccoli L., Pompa G., Brauner E. (2019). Timing for implant placement in patients treated with radiotherapy of head and neck. Clin. Ter..

[3] Kakarala K., Shnayder Y., Tsue T.T., Girod D.A. (2018). Mandibular reconstruction. Oral Oncol..

[4] Parise G.K., Guebur M.I., Ramos G.H.A., Groth A.K., da Silva A.B.D., Sassi L.M. (2018). Evaluation of complications and flap losses in mandibular reconstruction with microvascularized fibula flap. Oral Maxillofac. Surg..

[5] Etezadi A., Ferguson H., Emam H.A., Walker P. (2013). Multiple Remediation of Soft Tissue Reconstruction in Osteoradionecrosis of the Mandible: A Case Report. J. Oral Maxillofac. Surg..

[6] Darwich K., Ismail M.B., Al-Mozaiek M.Y.A.-S., Alhelwani A. (2021). Reconstruction of mandible using a computer-designed 3D-printed patient-specific titanium implant: A case report. Oral Maxillofac. Surg..

[7] O’dell K., Sinha U. (2011). Osteoradionecrosis. Oral Maxillofac. Surg. Clin. N. Am..

[8] Sandel H.D., Davison S.P. (2007). Microsurgical Reconstruction for Radiation Necrosis: An Evolving Disease. J. Reconstr. Microsurg..

[9] Raccampo L., Sembronio S., Tel A., Robiony M. (2023). Extended Complex Temporomandibular Joint Reconstructions Exploiting Virtual Surgical Planning, Navigation Assistance, and Custom-Made Prosthesis: A Comprehensive Protocol and Workflow. J. Pers. Med..

[10] Elledge R.O., Higginson J., Mercuri L.G., Speculand B. (2021). Validation of an extended total joint replacement (eTJR) classification system for the temporomandibular joint (TMJ). Br. J. Oral Maxillofac. Surg..

[11] Higginson J., Panayides C., Speculand B., Mercuri L.G., Elledge R.O. (2022). Modification of an extended total temporomandibular joint replacement (eTMJR) classification system. Br. J. Oral Maxillofac. Surg..

[12] Ângelo D.F., Prior A., Cardoso H.J. (2023). Postoperative Recovery after TMJ Arthroscopy: Masticatory Improvement and Postoperative Diet. Oral.

[13] van Gemert J.T., Abbink J.H., van Es R.J., Rosenberg A.J., Koole R., Van Cann E.M. (2018). Early and late complications in the reconstructed mandible with free fibula flaps. J. Surg. Oncol..

[14] van Baar G.J.C., Leeuwrik L., Lodders J.N., Liberton N.P.T.J., Karagozoglu K.H., Forouzanfar T., Leusink F.K.J. (2021). A Novel Treatment Concept for Advanced Stage Mandibular Osteoradionecrosis Combining Isodose Curve Visualization and Nerve Preservation: A Prospective Pilot Study. Front. Oncol..

[15] Baumann D.P., Yu P., Hanasono M.M., Skoracki R.J. (2011). Free flap reconstruction of osteoradionecrosis of the mandible: A 10-year review and defect classification. Head Neck.

[16] Nadella K.R., Kodali R.M., Guttikonda L.K., Jonnalagadda A. (2015). Osteoradionecrosis of the Jaws: Clinico-Therapeutic Management: A Literature Review and Update. J. Maxillofac. Oral Surg..

[17] Bedogni A., Bettini G., Bedogni G., Menapace G., Sandi A., Michelon F., Di Carlo R., Franco P., Saia G. (2021). Safety of boneless reconstruction of the mandible with a CAD/CAM designed titanium device: The replica cohort study. Oral Oncol..

[18] Sidebottom A.J. (2008). Guidelines for the replacement of temporomandibular joints in the United Kingdom. Br. J. Oral Maxillofac. Surg..

[19] Yoda T., Ogi N., Yoshitake H., Kawakami T., Takagi R., Murakami K., Yuasa H., Kondoh T., Tei K., Kurita K. (2020). Clinical guidelines for total temporomandibular joint replacement. Jpn. Dent. Sci. Rev..

[20] Gonzalez-Perez L.-M., Montes-Carmona J.-F., Torres-Carranza E., Infante-Cossio P. (2023). Total Joint Replacement for Immediate Reconstruction following Ablative Surgery for Primary Tumors of the Temporo-Mandibular Joint. J. Pers. Med..

[21] Mercuri L. (2012). Alloplastic temporomandibular joint replacement: Rationale for the use of custom devices. Int. J. Oral Maxillofac. Surg..

[22] Teschke M., Christensen A., Far F., Reich R.H., Naujokat H. (2021). Digitally designed, personalized bone cement spacer for staged TMJ and mandibular reconstruction—Introduction of a new technique. J. Cranio-Maxillofac. Surg..

[23] Suh J.D., Blackwell K.E., Sercarz J.A., Cohen M., Liu J.H., Tang C.G., Abemayor E., Nabili V. (2010). Disease relapse after segmental resection and free flap reconstruction for mandibular osteoradionecrosis. Otolaryngol. Neck Surg..

[24] Alakailly X., Schwartz D., Alwanni N., Demko C., Altay M., Kilinc Y., Baur D., Quereshy F. (2017). Patient-centered quality of life measures after alloplastic temporomandibular joint replacement surgery. Int. J. Oral Maxillofac. Surg..

